# Efficacy and Safety of Endoscopic Ultrasound-Guided Radiofrequency Ablation for Pancreatic Neuroendocrine Tumors: A Systematic Review and Metanalysis

**DOI:** 10.3390/medicina59020359

**Published:** 2023-02-14

**Authors:** Elia Armellini, Antonio Facciorusso, Stefano Francesco Crinò

**Affiliations:** 1 Gastroenterology Unit, Asst-Bergamoest, 24068 Bergamo, Italy; 2 Gastroenterology Unit, Department of Medical and Surgical Sciences, University of Foggia, 71122 Foggia, Italy; 3 Digestive Endoscopy Unit, University of Verona, 37129 Verona, Italy

**Keywords:** endoscopic ultrasound, pancreatic neuroendocrine tumors, insulinoma, radiofrequency ablation, RFA, pancreas, EUS-guided ablation, PanNETs

## Abstract

*Introduction*: The development of dedicated endoscopes and the technical evolution of endoscopic ultrasound (EUS) have allowed a direct approach to pancreatic neoplastic lesions both for diagnosis and treatment. Among the more promising targets are pancreatic neuroendocrine tumors (Pan-NETs). *Aim*: to describe the evolution of endoscopic ultrasound-guided radiofrequency ablation (EUS-RFA) with particular attention to the treatment of PanNETs, focusing on safety and clinical efficacy of the technique. *Methods*: MEDLINE, Scopus, and Cochrane Library databases were searched for studies reporting about EUS-RFA for the treatment of PanNETs. Studies with outcomes of interest were selected and results were reported to describe clinical success, complications, fol-low-ups, and electrodes used. Clinical success was defined as the disappearance of clinical symp-toms for functional (F-) PanNETs and as complete ablation per nonfunctional (NF)-PanNETs. The pooled data were analyzed by a random-effects model. *Results*: Nineteen studies were selected, including 183 patients (82 males, 44.8%) with 196 lesions (101 F-PanNETs and 95 NF-PanNETs). Pooled estimates for the overall AE rates for the clinical efficacy were 17.8% (95% CI 9.1–26.4%) and 95.1% (95% CI 91.2–98.9%) for F-PanNETs and 24.6% (95% CI 7.4–41.8%) and 93.4% (95% CI 88.4–98.4%) for NF-PanNETs. *Conclusions*: EUS-RFA appears to be a mini-invasive technique with a good safety and efficacy profile for the treatment of F- and NF-PanNETs. EUS-RFA could be of-fered as possible alternative to surgery for the treatment of low-grade NF- or F-PanNETs, especially for those patients that are not eligible or are at high-risk for surgery.

## 1. Introduction

Radiofrequency ablation (RFA) has been used for years for the treatment of solid malignancies, both with an intraoperative and percutaneous technique [1,2].

Pancreatic lesion ablation using thermal energy or through the injection of substances under EUS guidance is one of the recent applications of EUS [3,4]. EUS-RFA includes among the more promising targets PanNETs (others are pancreatic metastasis from renal cancer and pancreatic adenocarcinoma in the appropriate setting) [5,6].

**Figure 1:**
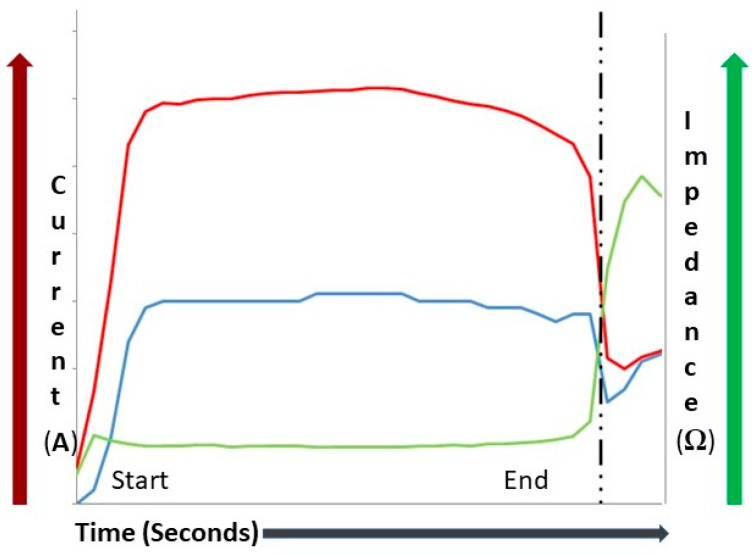
Basic principles of RFA (Courtesy of L. Portella).

The use of RFA in medicine is ubiquitous and dates back over 75 years. Energy produced from RFA has been used for the cautery of tissues or vessels and destruction of tumors or hypertrophic tissues, as well to ablate accessory conduction pathways in the heart to improve atrial fibrillation and other conditions. Ultrasound-guided percutaneous RFA has become one of the mainstays in treatment of hepatocellular carcinoma since the 1990s, as stated by Barcelona Clinic Liver Cancer (BCLC) guidelines, and it is used also for tumors of the lung, bone, prostate, and kidney [2]. Principles of RFA are explained in the following Figure 1 reproducing the relation between the main variables during RFA: change in Time (Seconds) of Current (Ampere, A), red line, and Impedance (Ohm, Ω), green line, at a constant Power (Watt, W), blue line.

Approaches to the treatment of pancreatic neoplastic lesions have been delayed because of the pancreas anatomy and of unsatisfactory results obtained by surgical or percutaneous treatments [7,8].

The evolution of EUS devices with the development of linear-array technology expanded the clinical utility of EUS. EUS guidance allows the precise placement of needles within focal mass lesions, hence therapeutic interventions represent a logical and inevitable advance in the development of EUS. The principal aim of this review is to provide a comprehensive overview on the main outcomes (safety and efficacy) of EUS-RFA for the treatment of pancreatic neuroendocrine tumors (PanNETs), both functional (F-) and non-functional (NF-).

## 2. Materials and Methods

**Figure 2:**
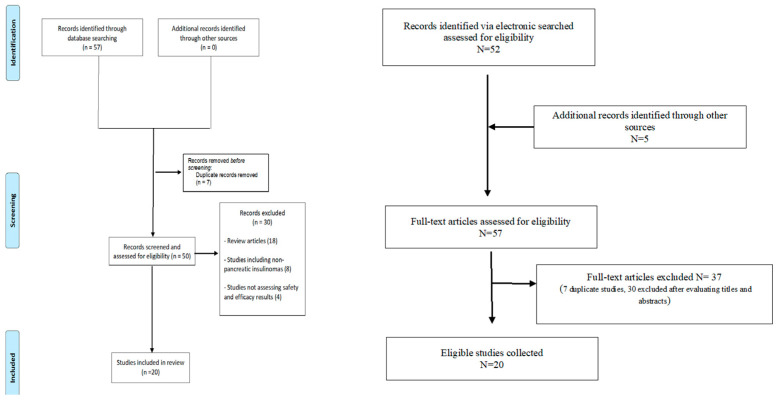
Study flow-chart.

This systematic review and metanalysis was performed in agreement with PRISMA guidelines [9]. MEDLINE, Scopus, and Cochrane Library databases were searched for studies reporting about EUS-RFA for treatment of PanNETs. The literature search was performed and verified by two independent reviewers (E.A. and A.F.) using the following index terms: “pancreatic neuroendocrine neoplasms” OR “pancreatic neuroendocrine tumors” OR “insulinoma” OR “PanNET” AND “endoscopic ultrasound-guided RFA” OR “EUS-RFA”. The inclusion criteria for studies were as follows: (1) Study subjects were adult (≥18 years old); (2) Prospective or retrospective study design, conference proceedings, case reports, and abstracts; (3) Reporting on safety and efficacy of EUS-RFA for the treatment of F- and NF-PanNETs; (4) The manuscript was written in English. The search included reports published until November 2022. A manual search of the reference lists of all review articles and primary studies retrieved was also performed. The flow-chart of the study is shown in Figure 2.

Studies with outcomes of interest were selected and results are reported to describe technical success, procedural details, adverse events (AEs) rate, and clinical efficacy.

Data were extracted independently and entered into standardized Excel spreadsheets (Microsoft Inc., Redmond, WC, USA). The following data were extracted from each study: first author; year of publication; number, gender, and age of patients; number of Pan-NETs; type of PanNETs (F- or NF-); tumor size (mean size in mm) and location; number of RFA sessions; clinical efficacy; occurrence and severity of AEs; and duration of follow-up.

Clinical success was defined as complete ablation (i.e., disappearance on cross-sectional imaging during follow-up) for NF-PanNETs and as disappearance of clinical symptoms for F-PanNETs. Safety was defined as the absence of AEs. AEs were defined and graded in accordance with an international lexicon [10].

Crude rates were extracted as outcome measures. Pooled estimates were obtained using a random-effects model because a high between-study variance in effect size was expected. Heterogeneity was assessed with the Pearson χ^2^ test and the I^2^ statistic.

## 3. Results

Fifty-seven potentially relevant studies were retrieved from the databases. Subsequently, seven duplicates and thirty studies were excluded after reviewing the titles and abstracts, as they were irrelevant articles and were not suitable for the research topic. Finally, we included 20 studies [5,11,12,13,14,15,16,17,18,19,20,21,22,23,24,25,26,27,28,29].

Overall, 183 patients (82 males, 44.8%) with 196 lesions (101 F-PanNETs and 95 NF-PanNETs) were included. Most of the cases were treated using a 18/19 G electrode-needle (EUSRA RF-electrode coupled with the VIVA RF generator, by Starmed, Taewoong Medical, South Korea) and a few cases were treated by a through-the-needle 1 Fr, monopolar, electrode-catheter (EndoHPB, Emcision UK, London, UK).

Diameters of the tumors widely varied between 4.5 mm and 30 mm and all but one F-PanNETs were insulinomas. Most of cases were treated by one single session; only a minority required multiple sessions (up to three). Follow-up time ranged between 1 and 41 months. Demographics, lesion features and the technical procedural details of F-PanNETs and NF-Pan NETs are summarized in Table 1 and Table 2, respectively.

**Figure 3:**
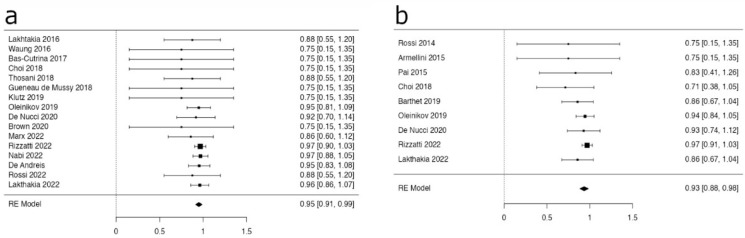
Forest plots reporting estimates for overall adverse event rates in F- (**a**) and NF- PanNETs (**b**). The pooled AE rates were 17.8% (95% CI 9.1–26.4%) and 24.6% (95% CI 7.4–41.8%) in F- and NF- PanNETs, respectively.

In the subgroups of F-PanNETs and NF-PanNETs, the pooled estimates for the overall AE rates were 17.8% (95% CI 9.1–26.4%) and 24.6% (95% CI 7.4–41.8%), respectively (Figure 3a,b).

The most common AEs were mild pancreatitis and abdominal pain. Overall, mild AEs were observed in 21/142 (14.7%) patients; moderate and severe AEs were observed in one patient each (0.007%).

**Figure 4:**
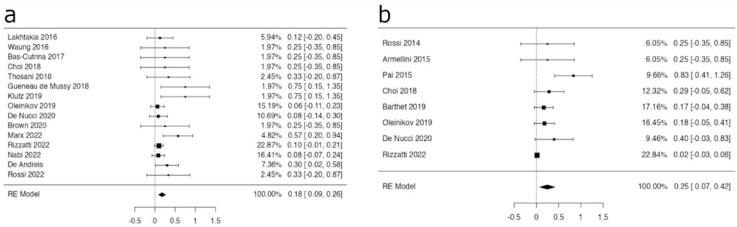
Forest plots reporting estimates for clinical efficacy of F- (**a**) and NF- PanNETs (**b**). The pooled efficacy rates were 95.1% (95% CI 91.2–98.9%) and 93.4% (95% CI 88.4–98.4%) in F- (**a**) and NF- PanNETs (**b**), respectively.

The pooled estimates for the clinical efficacy in the subgroups of F-PanNETs and NF-PanNETs were 95.1% (95% CI 91.2–98.9%) and 93.4% (95% CI 88.4–98.4%), respectively (Figure 4a,b).

## 4. Discussion

The term “radiofrequency” originates from the frequency of the electricity used which is like that of radio waves. RFA can be delivered by monopolar or bipolar electrodes. Monopolar electrodes have an insulated electrode needle with an active tip in connection to a grounding pad (passive electrode) placed on the patient’s body. It was the first developed model and has the advantage of deep penetration in tissues.

Bipolar electrodes use two active electrodes, adjacent at the tip of the needle; this characteristic implies less penetration and more limited volume of action. In both cases, the radiofrequency current translates into ion agitation within the surrounding tissue, which is converted by friction into heat and induces cellular death by means of coagulation necrosis. Energy transfer is dictated by Ohm’s Law: energy (J) = I^2^ × R × T (where “I” is the current, “R” is the tissue resistance (or properly impedance), and “T” is the time of application). Electrical energy is converted to thermal energy as resistance in the tissue is met [30].

Impendence depends on tissue hydration, electrolyte composition, collagen content, temperature, and other variables and becomes, in the end, the limiting step to the volume of the induced lesion. While energy is applied and radiofrequency acts, the charred tissue becomes an electrical insulator in the end, as shown by the rise in impedance. This phenomenon indicates inefficiency in further ablation (i.e., energy delivery) and marks the end of the procedure [30].

In 1999, Brugge et al. [31] reported the first experimental EUS-RFA in a pig model. They could easily foresee the possible application of ultrasound-guided therapies: “the development of endosonographically placed therapeutic devices may provide a unique alternative for the management of premalignant pancreatic lesions (e.g., islet cell tumors and cystic neoplasms) and potentially may offer palliative therapy for surgically unresectable malignant pancreatic tumors”.

These innovative ideas required ten years to come true. After Brugge’s first experimental model, years were needed to develop efficient EUS endoscopes and RFA devices, testing devices and accumulating experience on animal models in the meantime [32,33,34].

Finally, in 2008, a 2.2 mm needle-shaped bipolar hybrid cryotherm probe was used for experimental protocols in animal models [35,36] and humans [37] (Hybrid-therm, ERBE, Tubingen, Germany). These early experiences in patients with advanced pancreatic cancer showed that EUS-RFA could be applied in 16 patients (72.8%); amylase arose in 3 of 16 patients, and none had clinical signs of pancreatitis. Late complications arose in four cases: three were mostly related to tumor progression. A CT scan was performed on all patients, but only in 6 of 16 it was possible to clearly define the tumor margins after ablation.

In 2014, an Italian group reported treatment of a 9 mm NF-PanNET by EUS-guided monopolar RFA [11], preceding the description in 2015 by Pai et al. of the use of a monopolar “trough the needle” electrode for the treatment of cystic and solid lesions [12]. The device was the Habib EUS-guided RFA probe derived from the endovascular application concept: the Habib EUS-guided RFA probe (EndoHPB, Emcision UK, London, UK) was a 220 cm long, 1 Fr (0.33 mm), monopolar catheter that could be inserted through a regular 22-gauge FNA needle (Figure 2) and used in combination with commonly available radiofrequency generators. Its limitations are the limited penetration of energy and the small volume of tissue ablation.

In the same year, a water-cooled monopolar RFA needle with an active tip with different lengths was made commercially available after preliminary experiences on animal models [38].

Early experiences conducted between 2015 and 2016 offered satisfactory results in different fields of application ranging from pancreatic adenocarcinoma [39] to PanNETs [13,14] and extra-pancreatic solid lesions [40,41,42]. As we are writing this paper, the EUSRA RF Electrode is the only one available on the market. The concept of monopolar RFA that made the success of RFA in percutaneous ultrasound-guided procedure was translated into an EUS device by Taewoong Medical that produced the EUSRA RF-electrode. The volume of ablation corresponds to the length of the active needle, and the cooling system works to avoid fast charring of the tissues.

The EUSRA system is a monopolar radiofrequency needle consisting of a 19-gauge needle (18-gauge was the diameter of the first electrode-needle) with a 140 cm-long electrode lacking insulation over the terminal 5 to 20 mm tip, a dedicated RF current generator (VIVA RF generator) which allows control of power and impedance, and an inner cooling system intended to avoid fast tissue charring and to obtain large volume ablations circulating chilled saline through the needle.

Focusing on EUS-RFA of PanNETs, in 2015, a case of non-functioning PanNET (G2, diameter 20 mm, Ki 67 < 5%) fully ablated by a 10 mm EUSRA RF-electrode in one session with no complications and complete radiological response was reported [13]. In 2016, Lakhtakia et al. [14] treated three patients with symptomatic pancreatic insulinomas, not eligible for surgery. All had rapid symptom relief with biochemical improvement and remained symptom-free at 11–12 months of follow-up. There were no procedure-related adverse events.

Barthet et al. [5] in 2018 published a French prospective multicenter study aiming to assess the technical feasibility and safety of EUS-RFA for treatment of PanNETs and pancreatic cystic lesions. The study population included 12 patients that had 14 PanNETs (mean size 13.1 mm, range 10–20 mm). Among the 14 PanNETs, at 1-year follow-up, 12 had completely disappeared (86% tumor resolution), with three patients having a delayed response. After three complications (two pancreatitis and one small bowel perforation), a change in the original protocol reduced the complications rate: rectal diclofenac was used as recommended before endoscopic retrograde cholangiopancreatography (ERCP) to prevent post-endoscopic pancreatitis.

Antibiotic prophylaxis (2 g of amoxicillin and clavulanic acid intravenously) was used to prevent infection. At six months and one year, the success rates were 71.4 and 85.7, respectively.

J.A. Waung [15] in 2016 described successful treatment of an F-PanNET by the Habib needle; the procedure required multiple sessions to achieve a substantial response. Again, no complications were observed, and in 2017, Bas-Cutrina et al. [16] treated a small F-PanNET similarly.

In 2018, Choi [17] reported a single-center prospective study of seven NF-PanNETs and one insulinoma treated by EUSRA needle. Efficacy was reported in 75% after one year with mild adverse events in 25%. In the same year, Thosani et al. [18] described successful treatment of three F-PanNETs with 100% efficacy achieved at five months and Gueneau de Mussy et al. [19] and Kluz et al. [20] reported, respectively, the successful treatment of a 12 mm and a 9 mm F-PanNET.

In 2019, Oleinikov et al. [21] published a large retrospective series of cases treated in two tertiary centers in Israel to assess the feasibility, safety, and efficacy of EUS-RFA in a cohort of patients with F- and NF-PanNETs. The cohort included eighteen adults (eight women, ten men), aged 60.4 ± 14.4 years (mean ± SD), seven insulinoma patients, and eleven patients with NF-PanNETs. Twenty-seven lesions with a mean diameter of 14.3 mm (range 4.5 to 30) were treated. Technical success, defined as typical post-ablative changes on surveillance imaging, was achieved in 26 out of 27 lesions. Clinical response with normalization of glucose levels was observed in all (seven of seven) insulinoma cases within 24 h of treatment. Overall, there were no major complications 48 h post-procedure. No clinically significant recurrences were observed during follow-up (range 2 to 21 months).

In 2020, de Nucci et al. [22] published a prospective study of 10 consecutive patients with PanNETs ≤ 20 mm treated with EUS-RFA, and they were followed-up for at least 12 months. The mean size of the PanNETs was 14.5 mm (range 9–20 mm). Complete ablation of PanNETs was reached using a single-session RFA with a mean of 2.3 treatment applications per session. At both 6- and 12-month follow-ups, all lesions had disappeared on the CT scan. No major complications were observed. Brown et al. described the successful treatment of an 18 mm insulinoma in 2020 [23].

More recently, M. Marx et al. [24] analyzed the clinical course of patients with pancreatic insulinomas treated by EUS-RFA at two French tertiary referral centers. This study included seven patients, with a mean age of 66 years. EUS-RFA was feasible in all patients with immediate hypoglycemia relief after only one single treatment session; six of seven achieved a complete response by cross-sectional imaging and remained asymptomatic. Three patients had minor adverse events. One elderly patient developed a peripancreatic fluid collections and died consequently. This was a retrospective series collected over three years between 2017 and 2020, so it is difficult to ascertain if the significant complications rate and the serious adverse event were due to changes in operators, in the technique, or to other factors.

Finally, during the United European Gastroenterology Week 2022, the preliminary results of RAPNEN study [25] were shown in poster format. This large multicenter study included 62 patients treated from October 2019 to September 2022 at seven European centers. The results show a high rate of technical success and a good safety profile of the procedure.

In the same meeting, Lakhtakia et al. [26] reported a retrospective study of 26 patients, (mean age 49.3 ± 13.8, 12 males), 29 nodules, affected by F- and NF-PanNETs that were treated between 2014 and 2022 at the Asian Institute of Gastroenterology, Hyderabad, India. They reported significant improvement in mean fasting serum insulin (pre-RFA 39.4 ± 8.9 vs. post-RFA 13.1 ± 5.1; *p* < 0.001) and fasting blood glucose (pre-RFA 30.3 ± 7.3 vs. post-RFA 78.2 ± 7.7; *p* < 0.001) after a median follow-up of 31.5 months, and clinical response was seen in all the cases with insulinomas. In NF-PanNETs, significant responses on imaging were seen in 12 (85.7%) cases. Moreover, the same group [27] described the treatment of 12 patients affected by 15 insulinomas, obtaining a 100% clinical success.

In 2022, two papers by Borrelli de Andreis [28] (in press) and Rossi et al. [29] described the treatment of ten and three insulinomas, achieving complete clinical success with only mild complications in four cases.

In the reported experiences, EUS-RFA was used to treat both F- and NF-PanNETs.

The WHO 2019 classification morphologically distinguishes two groups of pancreatic neuroendocrine neoplasias (PanNENs): well-differentiated tumors or pancreatic neuroendocrine tumors (PanNETs) based on degree of differentiation and Ki67 index, further divided into PanNETs-G1 (Ki67 index ≤ 3%), PanNETs-G2 (Ki67 index between 4% and 20%), PanNETs-G3 (Ki67 index above 20%), and poorly differentiated neoplasms, so called neuroendocrine carcinomas (PanNECs) and mixed adenoneuroendocrine carcinomas (MiNen) [43].

The presence or absence of a hormonal hypersecretion syndrome further classifies PanNETs into functional and nonfunctional, which require different therapeutic approaches. However, which PanNETs should be treated by RFA and who should we treat? [44].

Most PanNETs (up to 70%) are nonfunctional, at least a half of which are asymptomatic and discovered on cross-sectional imaging requested for other reasons. International guidelines [45] suggest active surveillance for NF-PanNETs that are <2 cm and well-differentiated. Still, even in small PanNETs, the possibility of disease progression exists [46].

The traditional management of PanNETs has been surgical since excision of a potentially resectable lesion appeared to be a curative option. This is the treatment of choice in accordance with the Vienna Consensus Conference in 2016 [45], but limitations can be found in patient performance status, comorbidities, rate of progression, tumor size and grade, and in the non-negligible complication rate of the surgical option, even in cases of parenchyma sparing procedure [47,48,49,50,51]. However, the same guidelines define “a grey zone of hesitation” which encompasses patients affected by NF-PanNETs < 20 mm, G1-G2, where the choice between surveillance and treatment is affected by doubts and by the patient’s preference [52]. In these cases, the presence of disease may negatively affect the emotional well-being and become a substantial reason to favor treatment.

Addressing this topic, a time trends analysis conducted in an Italian high-volume center involving 587 patients from 1990 to 2015 showed that the number of resected PanNETs has increased in recent years, while the size (from 25 to 20 mm) decreased during the study period, but the G1 proportion (from 65% to 49%) still represented half of the resected tumors. After a mean follow-up of 75 months, recurrence analysis revealed that regardless of size, G1 NF-PanNETs with no nodal involvement and vascular invasion had a negligible risk of recurrence at 5 years [53]. Another interesting multicenter analysis showed how a surgical approach seems unnecessary for small G1 sporadic PanNETs [54]. Therefore, a mini-invasive option such as EUS-RFA appears desirable.

For F-PanNETs, the goal of EUS-RFA treatment is to reduce the hormonal hypersecretion syndrome with cessation of symptoms. This can be achieved by inducing necrosis of a sufficient amount of the secerning cells, and the complete ablation of the tumor is not required because of their very low malignant potential, although it would be advisable. On the contrary, for NF-PanNETs, the aim must be complete tumor ablation, with no vital neoplastic tissue left. Therefore, the definition, evaluation, technique, and effectiveness of the radiofrequency procedure vary in accordance with which type of PanNET is targeted.

This is a crucial aspect since the rate AEs may be influenced by the need to pursue complete ablation or, otherwise, if a partial debulking of the lesion is sufficient (i.e., partial ablation of a central area in a functioning tumor). In NF-PanNETs, the goal of complete ablation compels us to treat the margins of the tumor, and proximity to the normal pancreatic parenchyma potentially increases the risk of pancreatitis. In this setting, the availability of an RFA needle with different length active tips (EUSRA 19G needle has active tips ranging from 5 to 20 mm) and a careful selection based on pre-operative imaging and real time visualization is imperative for the success of the procedure and for abating adverse events.

For non-metastatic NF-PanNETs, the definition by which patients can benefit the most from RFA treatment is more complex than for F-PanNETs, as it is difficult to define which patients may benefit from careful follow-up or from surgical treatment, aiming to a balance between overtreatment (RFA/surgery in incidentally discovered PanNETs < 2 cm, that might not progress) and undertreatment (locoregional treatment in patients with undetected metastases).

It is not the aim of this review to discuss staging and prognostic factors of PanNETs, but exclusion of liver, lung, or lymph nodes metastases as well a careful evaluation of tumor grading, the Ki-67 proliferation index, and other prognostic factors, which are of paramount importance to indicate or rule out EUS-RFA [55,56,57,58,59,60,61,62,63,64].

A good guide for the selection of patients will probably be, in the future, the inclusion criteria indicated by the RAPNEN study, especially for NF-PanNETs: diameter of the lesion between 15 and 25 mm, ki-67 ≤ 5%, absence of distant metastases, symptoms, and inner calcifications. Considering functional lesions, multiple F-PanNETs do not look like a contraindication to EUS-RFA; on the contrary, treatment of multiple lesions in the same session appears feasible and this might become a further indication for EUS-RFA.

Our metanalysis showed pooled estimates for EUS-RFA clinical efficacy in the subgroups of F-PanNETs and NF-PanNETs of 95.1% (95% CI 91.2–98.9%) and 93.4% (95% CI 88.4–98.4%), respectively, which are consistent with a high rate of success in both categories. The subgroups of F-PanNETs and NF-PanNETs showed significant pooled estimate AE rates of 17.8% (95% CI 9.1–26.4%) and 24.6% (95% CI 7.4–41.8%), respectively, but notably, most of the AEs were mild (transient abdominal pain and mild pancreatitis) with a very limited incidence of severe AEs, and some of these were recorded among the earliest procedure of EUS-RFA. This should imply a good chance of improving these results in the future. Moreover, the alternative surgical treatment shows non-negligible rates of complications, even in cases of parenchyma sparing procedures, as reported in literature.

## 5. Conclusions

PanNETs are rare neoplasms with outcomes varying by stage, grade, and clinical presentation. A growth of PanNET incidence has been observed in the last few decades, with an increase in the low stage rate, as most patients are diagnosed at an initial stage of the disease. Therefore, a rise of the number of patients entering the “grey zone of hesitation” is expected.

About 70% of the PanNETs are nonfunctioning, and up to 50% are discovered in consequence of diagnostic imaging performed for other reasons, as asymptomatic lesions. International guidelines suggest active surveillance for small (1.5 to 2 cm), well-differentiated, asymptomatic, and NF-PanNETs, although they still entail the possibility of disease progression.

The need of an alternative approach to standard surgical treatment is obvious if we consider low-grade < 20 mm PanNETs, patients that are not eligible for surgery or are at high perioperative risk, or when a functional tumor requires debulking of hormone secreting cells. EUS-RFA appears to be a valuable alternative with high efficacy, a low adverse events rate, and major advantages such as low invasiveness and repeatability.

Moreover, the availability of different treatment options requires the involvement of different professional figures and shared decisions when therapeutic interventions are tailored in accordance with disease pathology, overall prognosis, and individual patient wishes.

Ongoing studies are expected to contribute to the final proof of concept and, in the long run, to define the appropriate indications for EUS-RFA.

## Figures and Tables

**Table 1:** Results from studies reporting the efficacy and safety of EUS-RFA for the treatment of F-PanNETs included in the meta-analysis **.

Study, Year	Study Design	N of Patients/ N of F-PanNET	Male/ Female	Age	Tumor Size (mean, mm)	Location	Device	N of RFA Sessions /Nodule	Efficacy *	Adverse Events	Follow-Up Months
Lakhtakia [14], 2016	Retrospective	3/3	M	45	19	H	19 G, Starmed	1	100	0	11.5
Waung [15], 2016	Retrospective	1/1	F	70	18	H	Habib EUS RFA	3	100	0	10
Bas-Cutrina [16], 2017	Retrospective	1/1	F	63	10	NR	Habib EUS RFA	1	100	0	10
Choi [17], 2018	Prospective	1/1	M	34	12	H	19G, Starmed	1	100	0	13
Thosani [18], 2018	Retrospective	3/3	NR	NR	NR	NR	NR	1.6 °	100	1	5
Gueneau de Mussy [19], 2018	Retrospective	1/1	F	69	12	T	19G, Starmed	1	100	1	2
Klutz [20], 2019	Retrospective	1/1	M	40	9	H	19G, Starmed	1	100	1	6
Oleinikov [21], 2019	Retrospective	7/7	4M, 3F	60.4	14.9	H:7, T:2	19G, Starmed	1	100	0	9.7
De Nucci [22], 2020	Prospective	5/5	2M, 3F	80	12.8	T	19 G, Starmed	1	100	0	12
Brown [23], 2020	Retrospective	1/1	F	66	18	H	19 G, Starmed	1	100	0	8
Marx [24], 2022	Retrospective	7/7	1M, 6F	66	13.3	H:1; T:6	19 G, Starmed	1	85,7	4	21
Rizzatti [25], 2022	Prospective	30/30	10M, 20F	61.9	11.9	H:12, T:18	19 G, Starmed	25:1; 4:2; 1:3	95,8	3	6
Lakhtakia [26]	Retrospective	12/12	NR	NR	NR	NR	19 G, Starmed	NR	100	NR	31.5
Nabi [27], 2022	Retrospective	12/15	7M/8F	46.7	NR	H:8T:7	19 G, Starmed	1: 10 >1:2	100	1	41
Borrelli deAndreis [28] in press	Retrospective	10/10	7M, 3F	11.9	11,9	H:3, T:7	19 G, Starmed	9:1,1:2	100	3	3
Rossi [29], 2022	Retrospective	3/3	2M, 1F	82.7	12	H:1, T:2	19 G, Starmed	1:2; 2:1	100	1	24

* Clinical efficacy was defined as disappearance of symptoms. ** table legend: N: number, H: pancreatic head, T: pancreatic tail, NR: not reported. ° in this case, a mean value was indicated.

**Table 2:** Results from studies reporting the efficacy and safety of EUS-RFA for the treatment of NF-PanNETs included in the meta-analysis **.

Study, Year	Study Design	N of Patients/ N of NF- PanNETs	Male/ Female	Age	Tumor Size (mean, mm)	Location	Device	N of RFA Sessions per Nodule	Efficacy *	N of Adverse Events	Follow-Up (Months)
Rossi [11], 2014	Retrospective	1/1	M	72	9	T	Habib EUS RFA	1	100	0	34
Armellini [13], 2015	Retrospective	1/1	M	76	20	T	18 G, Starmed	1	100	0	1
Pai [12],2015	Prospective	2/2	F	69.5	27.5	H	Habib EUS RFA	1:1,1:2	100	2	6
Choi [17], 2018	Retrospective	7/7	3M, 4F	56.1	20	H:2, T:5	19 G, Starmed	1:3, 2:2, 3:2	75	2	13
Barthet [5], 2018	Prospective	12/14	7M, 5F	59.9	13.1	H:3, T:11	19 G, Starmed	NR	86	2	12
Oleinikov [21], 2019	Retrospective	11/18	6M, 5F	65.8	14.2	H;11, T:7	19 G, Starmed	1	94.5	2	8.7
De Nucci [22], 2020	Prospective	5/6	3M, 2F	77.2	14.2	H:3, T:3	19 G, Starmed	1	100	2	12
Rizzatti [25], 2022	Prospective	32/32	22M, 10F	70.1	14.2	H:11, T: 21	19 G, Starmed	1:24, 2:7, 3:1	96.3	0	8
Lakthakia [26], 2022	Retrospective	14/14	NR	NR	NR	NR	19 G, Starmed	NR	85.7	NR	31.5

* Clinical efficacy was defined as complete disappearance of the lesion upon cross-sectional imaging during follow-up. ** table legend: N: number, H: pancreatic head, T: pancreatic tail, NR: not reported.

## Data Availability

The data presented in this study are available on request from the corresponding author.
